# Ageing with HIV: health implications and evolving care needs

**DOI:** 10.1002/jia2.25621

**Published:** 2020-09-30

**Authors:** Ahsan Ahmad, Malinee Neelamegam, Reena Rajasuriar

**Affiliations:** ^1^ Department of Medicine Section of Infectious Diseases AIDS Program Yale School of Medicine New Haven Connecticut USA; ^2^ Centre of Excellence for Research in AIDS (CERiA) University of Malaya Kuala Lumpur Malaysia; ^3^ Department of Epidemiology of Microbial Diseases Yale School of Public Health New Haven Connecticut USA; ^4^ Faculty of Medicine Department of Medicine University of Malaya Kuala Lumpur Malaysia

**Keywords:** ageing, HIV, PWH, WHO model of healthy ageing, interventions, life course

## 1 INTRODUCTION

At the end of 2019, there were an estimated 38 million people living with HIV (PLWH) globally and approximately 7.5 million were 50 years and older [1]. With improved access to antiretroviral therapy, these numbers are expected to increase rapidly, putting pressure on HIV health services to develop and implement programs which address the needs of an ageing population beyond traditional HIV care. PLWH experience an increased occurrence of chronic comorbidities including cardiovascular disease, poor bone health, metabolic abnormalities, frailty and neurocognitive decline [2].

The traditional approach to understanding the issues of ageing in PLWH, as demonstrated by many cohort studies, is focused on monitoring the presence of chronic comorbidities in older individuals. However, there are significant shortcomings to this approach, in that it is disease‐centred and focuses on measuring an individual’s health deficits at a stage in life where little can be done to prevent the decline in function. This approach also overlooks that the process of ageing with HIV is multidimensional, and in addition to the impact of the virus on biological systems, there are many other factors which shape the process of ageing, often well before an individual reaches middle age [3, 4]. To this end, WHO’s Model for Healthy Ageing provides a conceptual model which recognizes the interplay of all these factors and proposes a life course approach to ageing [5]. This model establishes a unique framework to measure individualized health in PLWH who experience an increased lifespan with antiretroviral therapy, but not necessarily in their “healthspan” [6].

The *WHO Model of Healthy Ageing* describes ageing as a holistic process that enables individuals to be and to do what they have reason to value throughout their lives. The model encourages policy makers and health professionals to look beyond disease states and approach ageing as a process to maximize an individual’s functional ability. This is achieved by optimizing two components throughout an individual’s adult life: their intrinsic capacity (IC) which is made up of five domains (cognition, mobility, psychological health, vitality and sensory) and the external environment which encompasses all the factors which contribute to the context of their daily lives, that is the social determinants of health [7]. There is a strong interplay between these two components which is often overlooked in our clinical approach to ageing, in that the external environment can help balance and compensate for losses in IC and help maintain function. This is crucial in the context of PLWH, who may have less control over how HIV impacts biological systems which compromise the domains of IC, but may have more opportunity to change aspects of their daily environment. These components come together to define an individual’s *health trajectory*—a dynamic variable of ageing.

**Figure 1:**
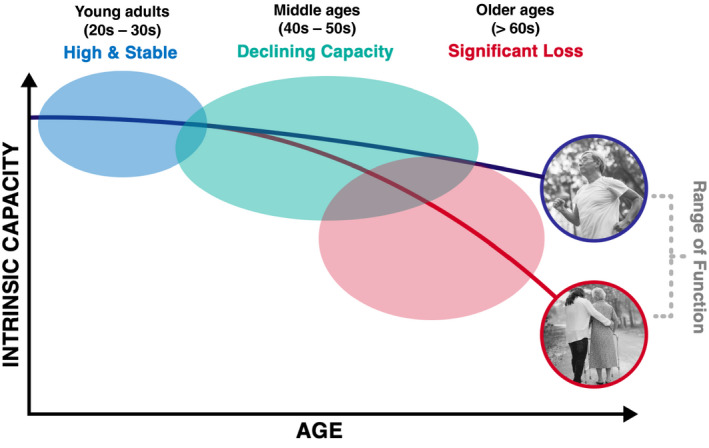
The three levels of intrinsic capacity throughout the adult life course as depicted in WHOs model for Healthy Ageing.

WHO has proposed three stages of this trajectory (Figure 1); a period of high and stable capacity usually corresponding to early adult life, followed by a transition to declining capacity in middle life and subsequently significant loss in capacity in later life which is associated with frailty and disability [6, 8]. This staging may serve as a framework to help redefine how we view the process of ageing in PLWH. For example young adults with perinatally acquired HIV in their 20s are not chronologically old. Yet, there are reports of cognitive impairment, declines in vitality and mental health issues in these young PLWH [9] which compromise their ability to function optimally within this stage of life. This may also have long‐term implications on health trajectories in their later life. The process of ageing with HIV has not been studied to understand the transitions in function across the adult life course and almost never includes individuals in the 20s or 30s. The WHO model also encourages the inclusion of social analysis into clinical practice by examining the external environment encountered from living with HIV. PLWH are often stigmatized, marginalized and discriminated against, and this impacts access to and utilization of healthcare, financing health care needs, employment opportunities and social support networks. Each of these challenges have different implications at each stage of the health trajectory. Addressing social needs should be viewed as an opportunity to preserve function in PLWH. Even at later stages, evidence from the HIV‐negative population indicate that an individual’s day to day environment, if supportive, can help slow the decline in function [10].

The WHO framework for Healthy Ageing recommends several evidence‐based behavioural interventions to address age‐related decline in IC. These include multimodal exercises, supplemental nutrition, medication review, sensory aids and structured cognitive and psychological therapy mechanisms [11]. Although these were initially investigated to address age‐related health decline of older adults without HIV, they can be modified and applied to address similar concerns among PLWH.

There is still a pressing need for a framework of action, to shift the HIV care paradigm to include implementable healthy ageing interventions across the life course. Monitoring of individual capacities as markers of ageing over the lifespan of PLWH should be prioritized, to ensure the earliest possible implementation of interventions to reduce and prevent premature functional decline. Additionally, strategies such as identification and treatment of clinical comorbidities, substance use disorders, and medication reviews, which are more readily actionable in clinical settings, should be optimized [12]. Empowerment of PLWH to advocate for their needs and to actively participate in the management of their non‐HIV‐related comorbidities is essential. Shared‐decision making models can empower and actively engage PLWH to effectively manage their unique healthcare and social needs as they age [13]. Evidence‐based mobile‐health and machine‐learning strategies can also be leveraged to include healthy ageing interventions to deliver a more comprehensive and integrated care plan for PLWH.

Ageing is asynchronous and heterogenous in nature, and approaches which use chronological age to define issues of ageing in PLWH are inadequate. More studies on health trajectories of PLWH with health defined as a composite of physical, mental and functional capacities are needed. These studies will be integral to developing policies surrounding the social needs of PLWH and to strengthening HIV care models which ensure the process of healthy ageing in PLWH.

## COMPETING INTERESTS

Authors do not have conflicts of interests/ competing interests to declare.

## AUTHORS’ CONTRIBUTIONS

Authors (alphabetically): Ahsan Ahmad (AA), Malinee Neelamegam (MN) and Reena Rajasuriar (RR) contributed equally to this viewpoint. This viewpoint was developed to reflect the salient points of the plenary session “Growing up and growing old with HIV: Health implications and evolving care needs” presented by RR at the 23^rd^ International AIDS Conference Virtual, 6‐10 July 2020.

## AUTHORS’ INFORMATION

Ahsan Ahmad is a Research Associate at the Department of Medicine, Section of Infectious Diseases, AIDS Program at the Yale School of Medicine and a researcher at the Centre of Excellence for Research in AIDS (CERiA), at University Malaya, Faculty of Medicine. Malinee Neelamegam is a Global Health Equity Scholar at the Yale School of Public Health, and a researcher at the Centre of Excellence for Research in AIDS (CERiA) at University Malaya, Faculty of Medicine. Reena Rajasuriar is an Associate Professor at the Department of Medicine at the Faculty of Medicine at University Malaya, and Principal Investigator at the Centre of Excellence for Research in AIDS (CERiA), University of Malaya, Kuala Lumpur.

## ABBREVIATIONS

IC, Intrinsic Capacity; PLWH, Persons with HIV; WHO, World Health Organization.

## References

[1] United Nations Department of Economic and Social Affairs (UNDESA) . World Population Report Highlights 2019 [cited 2020 Aug 18]. Available from: https://www.un.org/en/development/desa/population/publications/pdf/ageing/WorldPopulationAgeing2019‐Highlights.pdf

[2] Guaraldi Giovanni , Milic Jovana .The interplay between frailty and intrinsic capacity in aging and HIV infection. AIDS Res Hum Retroviruses. 2019 1013–22. 10.1089/AID.2019.0157 31452380

[3] Kohanski RA , Deeks SG , Gravekamp C , Halter JB , High K , Hurria A , et al. Reverse geroscience: how does exposure to early diseases accelerate the age‐related decline in health? Ann N Y Acad Sci. 2016;1386(1):30–44. 27907230 10.1111/nyas.13297

[4] Rajasuriar R , Chong ML , Ahmad Bashah NS , Abdul Aziz SA , Mcstea M , Lee ECY , et al. Major health impact of accelerated aging in young HIV‐infected individuals on antiretroviral therapy. AIDS. 2017;31(10):1393–1403. 10.1097/QAD.0000000000001475. 28358731

[5] World Health Organization (WHO) . World Report on Ageing and Health. 2015 [cited 2020 Aug 18]. Available from: https://apps.who.int/iris/bitstream/handle/10665/186463/9789240694811_eng.pdf?sequence=1

[6] Beard JR , Officer A , de Carvalho IA , Sadana R , Pot AM , Michel J‐P , et al. The World report on ageing and health: a policy framework for healthy ageing. Lancet. 2016;387(10033):2145–2154. 10.1016/S0140-6736(15)00516-4. 26520231 PMC4848186

[7] Cesari M , Araujo de Carvalho I , Amuthavalli Thiyagarajan J , Cooper C , Martin FC , Reginster J‐Y , et al. Evidence for the domains supporting the construct of intrinsic capacity. J Gerontol A Biol Sci Med Sci. 2018;73(12):1653–1660. 10.1093/gerona/gly011. 29408961

[8] Belloni G , Cesari M . Frailty and intrinsic capacity: two distinct but related constructs. Frontiers in Medicine. 2019;6:133. 31275941 10.3389/fmed.2019.00133 PMC6591451

[9] Abrams EJ , Mellins CA , Bucek A , Dolezal C , Raymond J , Wiznia A , et al. Behavioral health and adult milestones in young adults with perinatal HIV infection or exposure. Pediatrics. 2018;142:e20180938 10.1542/peds.2018-0938. 30097528 PMC6317560

[10] Michel JP , Sadana R . "Healthy Aging" concepts and measures. J Am Med Dir Assoc. 2017;18(6):460–464. 10.1016/j.jamda.2017.03.008. 28479271

[11] Thiyagarajan JA , Araujo de Carvalho I , Peña‐Rosas JP , Chadha S , Mariotti SP , Dua T , et al. Redesigning care for older people to preserve physical and mental capacity: WHO guidelines on community‐level interventions in integrated care. PLoS Med. 2019;16:e1002948. 31626651 10.1371/journal.pmed.1002948 PMC6799894

[12] Erlandson KM , Karris MY . HIV and aging: reconsidering the approach to management of comorbidities. Infect Dis Clin North Am. 2019;33(3):769–786. 10.1016/j.idc.2019.04.005. 31395144 PMC6690376

[13] Okoli C , Brough G , Allan B , Castellanos E , Young B , Eremin A , et al. Shared decision making between patients and healthcare providers and its association with favorable health outcomes among people living with HIV. AIDS Behav. 2020;1–12. 32748158 10.1007/s10461-020-02973-4 PMC7397451

